# Towards a Link between Quantitative and Qualitative Sciences to Understand Social Systems Using the Example of Informal Settlements

**DOI:** 10.3390/e25020262

**Published:** 2023-01-31

**Authors:** John Friesen

**Affiliations:** Chair of Fluid Systems, Technische Universität Darmstadt, 64287 Darmstadt, Germany; john.friesen@fst.tu-darmstadt.de; Tel.: +49-6151-16-27103

**Keywords:** informal settlements, slums, slum-modeling, social modeling, self-organization

## Abstract

Urbanization is one of the defining trends of our time and appropriate models are needed to anticipate the changes in cities, which are largely determined by human behavior. In the social sciences, where the task of describing human behavior falls, a distinction is made between quantitative and qualitative approaches, each of which has its own advantages and disadvantages. While the latter often provide descriptions of exemplary processes in order to describe phenomena as holistically as possible, the goal of mathematically motivated modeling is primarily to make a problem tangible. Both approaches are discussed in terms of the temporal evolution of one of the dominant settlement types in the world today: informal settlements. These areas have been modeled in conceptual works as self-organizing entities and in mathematical works as Turing systems. It is shown that the social issues surrounding these areas need to be understood both qualitatively and quantitatively. Inspired by the philosopher C. S. Peirce, a framework is proposed in which the various modeling approaches describing these settlements can be combined to arrive at a more holistic understanding of this phenomenon by using the language of mathematical modeling.

## 1. Introduction

The Bible, one of the most influential books of the last two millennia [1], begins its story in a garden and ends it in a city. In this way, it describes a development of mankind that originated with sedentarization and reached its provisional climax in 2008 with more than half of humanity living in cities. This trend is likely to intensify in the coming decades [2]. The urban population is expected to continue to increase, with about 90% of this increase occurring in South Asia and sub-Saharan Africa.

Cities are drivers of innovation as well as being economic, political, and cultural centers offering their inhabitants both opportunities and risks [3]. These very broad-brush thoughts motivate the diverse work that has been undertaken in the field of urban studies in recent decades. Since cities are an integral part of the reality of human life, scientists with the perspectives of different domains seek to understand them by asking: What are cities? How do they function?

The urban researcher Juval Portugali [4], following the well-known thesis of the writer and scientist C. Snow, divides the research on cities into two cultures: One from the humanities (i) and one from the natural sciences (ii). On the one hand (i), there are the well-known qualitative works from the social sciences describing to what extent cities shape our lives: from Georg Simmel’s *The Metropolis and Mental Life* [5] and Wirth’s *Urbanism as a way of life* [6] to recent works such as Ananya Roy’s work on informality [7] or Richard Sennett’s *Building and Dwelling* [8]. On the other hand (ii), more quantitative approaches inspired from the natural sciences were introduced at the same time. Auerbach’s work on the size distribution of cities [9], followed by the work of G.K. Zipf [10] leading to the famous Zipf’s Law, showing that the size and frequency of cities are linked and Christaller’s *Central Place Theory*, highlighting the spatial distribution of different city types within a region [11], are examples for this so called second culture. Modern examples for quantitative models of cities are the works of Michael Batty [12,13,14]. These and many other scholars from various disciplines with different perspectives have attempted to understand, describe, and model *the city* in the last century, reflecting the fact that urbanization is a process that reaches into, shapes, and is shaped by almost all areas of life.

Quantitative and qualitative studies in the social sciences both aim to understand and describe human behavior as comprehensively as possible. However, they differ in their goals and methods. Quantitative studies often seek to identify causal relationships and use statistical methods to test hypotheses, while qualitative studies aim to gain a deeper understanding and insight into the subjective experiences of individuals and groups. Both types of approaches are used to address societal problems and both involve constructing models of reality. As sociologist Max Weber noted, the goal of the social sciences is often to understand human behavior, while the goal of the natural sciences is often to explain it causally [15]. Both types of approaches seek this knowledge in order to use it to solve societal problems [15] and both do it by building models of the reality.

However, what are the pressing societal problems and what kind of models—*quantitative or qualitative*—are appropriate to provide solutions?

In line with the above motivation, the question of appropriate modeling approaches based on pressing issues in cities is discussed in this paper. The focus is on informal settlements, since this type of settlement is the major mode, in which urbanization takes place in the 21st century [2]. Using this example, it is shown that neither a purely quantitative nor a purely qualitative approach alone is sufficient. Therefore, a framework to combine both views in a holistically way is proposed.

To do so, Section 2 presents in a general way why dealing with informal settlements and investigations of their temporal development is important. In Section 3, the usefulness of certain models in describing the temporal behavior is discussed, and the motivation for the use of computational social sciences is provided. In Section 4, a framework on how quantitative and qualitative approaches can be combined using mathematical modeling to gain an in-depth understanding of a social system is developed. The approach is explained using the example of informal settlements.

## 2. Informal Settlements

**Figure 1:**
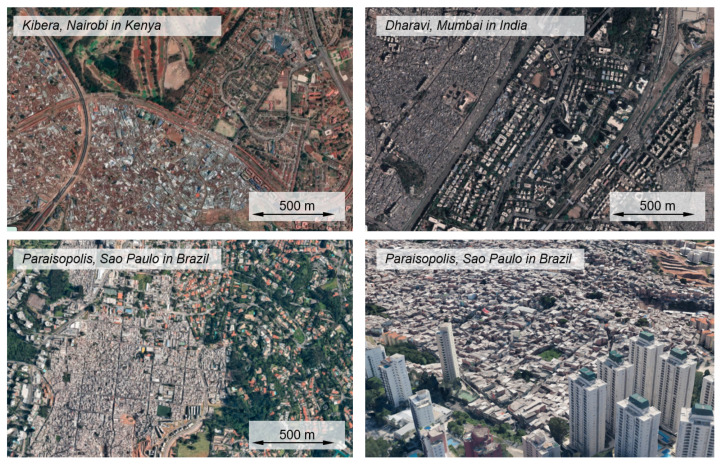
Three of the most famous informal settlements: Kibera, Nairobi in Kenya, Dharavi, Mumbai in India and Paraisopolis, Sao Paulo in Brazil. Maps data: 2023 Google (all figures), Airbus, Maxar Technologies (Kibera and Dharavi).

The aforementioned process of urbanization has been linked to segmentation from the beginning. There is not just one city, with a homogeneous structure, but the city itself is fragmented [6]. Different social groups live in different areas of the city [6]. Plato already made this observation in *The republic* by dividing the city in one city of the poor, and one of the rich. When looking at modern cities of the Global South, such as Nairobi in Kenya, Mumbai in India, or Sao Paulo in Brazil (Figure 1), this observation still holds. Next to expensive high-rise apartments, there are settlements with corrugated iron huts, whose inhabitants are disadvantaged in many aspects. The lack of access to infrastructure observed in these areas leads to an increased likelihood of the spread of infectious diseases or occurrence of accidents, economic disadvantage and, as a result, greater psychological distress for the population [16,17,18,19]. These areas are called slums, informal settlements, or deprived areas, although local terms such as *favela*, *barrio*, or *bandung* are often used as well. In the following, the terms slums and informal settlements will be used synonymously.

According to the United Nations, currently over one billion people, or one in eight persons, are living in slums. This number is expected to increase until 2030 up to two billions [2]. It is therefore of upmost importance to understand these types of settlements, especially for the following reasons: to learn how the population living there behaves socially [20], how it manages to deal with obstacles and dangers, and how it emerges strengthened from crises [21]. The last point is of particular interest, since informal settlements are often subject to crises, such as landslides, due to their often exposed locations [18]. Besides, the mode of sustaining life in the city through a kind of self-organization almost without institutional support is of utmost interest [22].

Additionally, planners and (local) decision-makers also need an understanding of how this kind of settlement will behave temporally, and where and in which places the estimated additional one billion inhabitants will settle to provide basic infrastructures for water, sanitation, or healthcare [16].

Therefore, Mahabir et al. [23] proposed a framework to conceptualize and contextualize slums. Their aim is to first develop a generic concept of informal settlements and then adapt it to the respective observations in specific cities. This in turn leads to new conceptualizations, resulting in an iterative process of understanding this kind of settlements. Roy et al. [24] or Hofman et al. [25] analyze different models and distinguish between different modeling types, such as celluar-automata or agent-based models to describe the emergence and temporal development of informal settlements. Examples are the cellular-automata model of Badmos et al. [26], describing slum growth in Lagos, Nigeria, or the agent-based model of Roy and Lees [27] to understand the resilience of slums in Bangalore, India.

The provision of infrastructures for the inhabitants of these settlements is of utmost importance to the global community and is addressed in various of the Sustainable Development Goals [16]. Therefore, for local authorities and planners one important question related to the aforementioned question of what a city is and how it functions is how cities and especially informal settlements develop? Do they grow? Do they shrink? Do they stay constant? Which kind of mechanism is causal for their changes?

This circumstance leads us to the central tension between qualitative and quantitative approaches addressed at the beginning of this paper. On the one hand, informal settlements are areas that differ from their surroundings by their morphology [28] and can thus be analyzed with quantitative statistical methods [29,30,31]. On the other hand, the development of these settlements cannot be described without analyzing the human behavior of their inhabitants, and future informal areas cannot be predicted if no models of this behavior are known. In order to understand the development of these areas, insight into the behavior of their inhabitants must be gained. To provide adequate infrastructures for informal settlements, models of possible future development of these areas are needed. Informal settlements are in this sense socio-technical systems in which both the social and technical aspects must be considered. Thus, the question of what kind of model best suits to answer these questions remains.

## 3. Modeling Approaches for Social Systems

To form models of the reality surrounding us in order to be able to deal with it in an adequate way is a basic trait of being human. This can be easily illustrated by the example of the movement models formed by our cerebellum [32] to provide systematic sequences of muscle contractions for an intended action. The goal of any model building is to represent the relevant aspects of reality in an appropriate way, to neglect the non-relevant ones and to describe the links and the interplay between them. The more aspects are included in the respective models, the more detailed questions can be answered [33]. The central question that therefore arises is which part of reality is relevant and which is negligible for a specific model.

There is a discussion in the social sciences that models are becoming increasingly complex. Healy [34] points out, that instead of incorporating more and more nuances in sociological models and describing the possible influences of these nuances on the outcome, models should be as simple as possible. This quest for simple models is particularly useful in the context of informal settlements, as these areas are examples of data scarce environments.

However, the discussion of which type of method is best suited to describe sociological phenomena is not only limited to questions related to urban sciences or urban studies, but can also be conducted on an even more general level. Again and again in recent years, the desire has been expressed to combine the views of different scientific perspectives, such as the natural sciences and the humanities [4]. While the latter often provides qualitative descriptions of exemplary processes in order to describe phenomena as holistically as possible, the goal of mathematically motivated modeling is primarily to point to a problem in a quantitative, concrete, and tangible way. A comprehensive list of quantitative and qualitative methods in social sciences and their comparison can be found in Ref. [35]. In the following, a short summary of these is given.

### 3.1. Qualitative Research

Smith [36] clearly points out, that, according to Dilthey, the advantage of qualitative models in social sciences is the focus on the individual and its values, emotions, and subjectivity. Relating this to the growth of informal settlements, a qualitative investigation could ask the question of what reasons motivated residents to move there. In qualitative research, the motivation of people can be described in narratives and concepts, yielding to a deeper understanding of the migration process. This can be helpful for decision makers to have concrete narratives and the related societal issues in mind in order to plan appropriate actions.

Although in a next step, it would also be possible to quantify the possible reasons for migration, the formation of the classes itself is a qualitative task in nature. This classification in turn depends on the researcher conducting it. Since in this case humans seek to understand the behavior of other humans, there is no *context-free or neutral scientific language* to describe sociological phenomena. For this reason, Max Weber points out, that it is different when researchers observe the behavior of an atom or the desires and intentions of human beings [36]. Connected with this aspect is the disadvantage of this type of research. It is often not clear how representative the respective narrative is for a given overall situation. Nor can the results be used to plan specific actions, such as infrastructure implementation.

### 3.2. Quantitative Research

In contrast, quantitative data-based approaches do not focus on qualitative descriptions, but on the empirical observations of reality. In addition to descriptive methods (e.g., regression) [35], mechanistic works in particular have been established in recent years to model different scenarios of the behavior of social systems. They are referred to in the following. Holme and Liljeros [37] provide a comprehensive overview of the history of computational models in social sciences. They summarize the use of simulation models in the natural sciences, beginning with the well-known Monte-Carlo simulations of John von Neumann over approaches in complexity research and game theory up to network theory. They show how computable models were developed around the same time in operations research, political science, linguistics, economics, behavioral science, or geography. Unlike qualitative models, these computational models have the advantage of the possibilities to verify theses, analyze emergent phenomena, and perform forecasting [37]. The famous meteorologist Lewis F. Richardson, who developed an equation-based theory to describe the emergence, or rather the buildup of a conflict between two nations as a dynamic system using ordinary differential equations, formulated his favor for mathematical and quantitative models in the following statement [38]:

*Another advantage of a mathematical statement is that it is so definite that it might be definitely wrong; and if it is found to be wrong, there is a plenteous choice of amendments ready in the mathematicians’ stock of formulae. Some verbal statements have not this merit; they are so vague that they could hardly be wrong, and are correspondingly useless.*

Although Richardson was aware of the multitude of qualitative descriptive works on peace and conflict studies, he models this complex problem using equations, examines them through stability analysis, derives hypotheses, and investigates them mathematically [39].

Based on this tradition and in the context of the emergence of Big Data, in the beginning of the 21st century, there was a call in *Science Magazine* for quantitative formulations of questions in social science [40], not only related to urban science. Unfortunately, the authors observed after 10 years, that these attempts of integrating computational models into the social sciences is not very common [41]. Although manifold possibilities are seen to obtain the most objective representation of reality by applying mathematical methods, critics point out that by attributing human behavior to quantitative descriptions, human narratives and therefore a core of being human are relegated [42].

## 4. Combination of Qualitative and Quantitative Sciences

**Figure 2:**
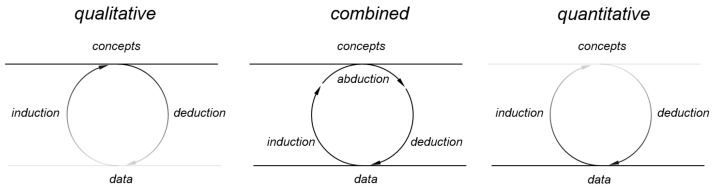
Different concepts of research. Adapted from Pelz [33] and Friesen [43].

As already described above, research approaches can take different forms (cf. Figure 2). Besides the distinction between quantitative and qualitative sciences, the one between inductive and deductive research has been referred to in lectures or seminars on the philosophy of science. While in deduction a general principle is related to a specific observation, in induction an observation is used to infer a rule. Following Pelz [33], induction and deduction describe ways to move from the world of concepts to the socio-technical world that can be described by data, and vice versa. In qualitative as well as in quantitative research both ways to conduct research are common. Nevertheless, as shown above, the focus of qualitative science often relies on concepts while quantitative science highlights data [36].

In the following, it is shown how qualitative models of a sociological (or more precisely socio-technical) phenomenon, namely informal settlements, can be used as a starting point for analysis and how mathematical models can be generated based on them. In the following step, the results of these models can be fed back into the social sciences using modern techniques such as data driven modeling, leading to a more thorough understanding of this settlement type.

The aim is to bring the two approaches (qualitative and quantitative) closer together by introducing Peirce’s concept of the inferential triad. The science theorist Charles Sanders Peirce (1839–1914) investigated the question on how new concepts or ideas are developed in science. In addition to the concepts of induction and deduction already mentioned above, he introduced *abduction*, which is considered *the process of forming an explanatory hypothesis. It is the only logical operation which introduces any new idea* [44].

Instead of focusing on either concepts or data, the goal is an iterative process of matching concepts with empirical evidence and to develop new approaches from observations or surprising facts. These surprising facts then lead to a new theory explaining their appearance, similar to Thomas Kuhn’s often cited concept of paradigm shifts [45].

In sociology or the social science, approaches combining the different concepts are called mixed methods or triangulation. Denzin, who is considered to be one of the first researchers to systematically explore these types of methods in the 1970s [46] stated that *The use of multiple methods, or triangulation, reflects an attempt to secure an in-depth understanding of the phenomenon in question. Objective reality can never be captured. We only know a thing through its representations* [47]. Therefore the basic idea behind triangulation is to combine different kinds of methods, qualitative and quantitative ones, in order to obtain a picture of the problem at hand as holistic as possible. The proximity of this approach to the concept of the inferential triad by Peirce is a well known fact [48].

While in the latter works mainly quantitative and qualitative approaches from the social sciences are combined, the aim in the concept presented here is to include approaches and concepts from the natural sciences as well.

In the following subsections an example of how such kind of inferential triad can be applied in the study of informal settlements to gain a more holistic understanding of this phenomenon is presented.

### 4.1. Autopoiesis, Self-Organitzation and Open Systems in Sociological Concepts

In sociology, two of the the best known approaches to describe social systems are the system theories of Talcott Parsons and, building on this, Niklas Luhmann. While Parsons focuses mainly on the structures of social systems, Luhmann’s focus is on the functions that have to be fulfilled within them. To describe the change of social systems, Luhmann resorts to the term *autopoesis*. This Greek term describes something similar to *self-creation*. It was introduced by Maturana and Varela [49] as a characteristic of living systems. Maturana and Varela describe organisms as autopetical systems that exhibit operational coherence, i.e., run according to certain principles and change according to stimuli that act on them from the outside. Autopoesis is therefore a famous concept of self-organization. As mentioned, Niklas Luhmann takes up this approach and applies it to social systems [50]. Following the work of Maturana and Varela, he describes social systems as creating themselves in an autocatalytic process using the phrase *order out of noise*, referring to the observation that structures emerge through self-organization [50]. Central is the idea that these systems are operationally closed, as in they obey certain rules, but are constantly in contact with their environment. Luhmann’s concept was critically discussed and it was pointed out that although it is interesting as an explanatory model, his transfer of Maturanas and Varelas model was not concrete and thus not verifiable and falsifiable [51].

Another recent sociological work describes cities as *open systems*. In his work "Building and Dwelling," Richard Sennett characterizes different aspects of an open city and explicitly draws on models of self-organization [8].

**Figure 3:**
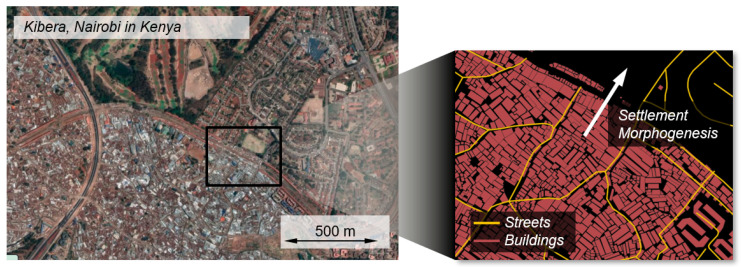
Schematic figure of the morphogenesis of Kibera, Nairobi, adapted from Dovey [22]. Maps data: 2023, Google, Airbus, Maxar Technologies.

The principle of self-organization is often spoken of, not only in relation to cities in general, but also to informal settlements in particular [52,53,54,55]. One very recent approach in this context is the work of the architect Kim Dovey and colleagues. They describe an informal settlement not as a name for a specific area, but as a process and production mode using the term informal settlement to explain how certain areas in cities behave over time [22]. With his co-authors he uses the term *morphogenesis* to create a connection between informal settlement and the morphogensis of an organism. The analogy between the temporal development of a road network within an informal settlement and the angiogenesis in an organism in their work is striking (see Figure 3).

That the concepts of autopoesis, open systems and self-organization used here are strongly inspired by quantitative sciences such as thermodynamics or biology is usually clear to the authors of the previous works. However, the question arises why the mentioned works have resorted to the concepts, but a quantitative mathematical modeling has failed to take place [56,57]. If one refers back to the quote by Richardson mentioned before, one could provocatively ask whether qualitative models are not useless, since their truth content cannot be verified and their use to develop scenarios for, e.g., infrastructural provision, is unknown.

### 4.2. Mathematical Models of Self Organization and Their Application to Social Systems

Physicists, chemists, or engineers have a different approach to concepts of self-organization. Through their training in thermodynamics, they are able to transfer these concepts into quantitative models, as Rapaport described in his critique of Richardson’s article [58]:

*A mathematical model is more characteristically a point of departure rather than a point of arrival in the construction of a theory. In this way it is akin to the null hypothesis, which, incidentally, also often involves the construction of a mathematical model. In most cases, null hypotheses are made so that they can be refuted. As a by-product of the refutation of the null hypothesis, biases are usually discovered which point to the direction of search for causes; It is much the same with mathematical models. These models are often deliberately made simple-minded, with full knowledge that they do not represent reality. Their chief value is that they lead to compelling consequences. These consequences are then compared with observations. As often as not, the derived consequences do not agree with the observations. However, then the direction and magnitude of the departures may indicate the direction of further search.*

Ilya Prigogine was one of the first to describe self-organization using thermodynamic models. In his work on *dissipative structures* he focused on open systems and their behavior [59]. The possibility of considering cities as open systems has also been seen by himself. He explicitly referred to cities as dissipative structures in different ways and the related questions were investigated by his co-worker Peter Allen [60,61]. In recent years, quantitative models to describe self-organization have been applied to cities. For example, Levashova [62] describe the development of the megacity Shanghai, China through self-organization using a reaction-diffusion model.

In the same year, Pelz et al. [63] applied a similar approach to describe the emergence of informal settlements in different cities of the Global South based on the mathematical model of morphogenesis, presented by Alan Turing in 1952 [64]. Instead of focusing on a qualitative description of the process, Turing developes in his seminal paper *The chemical basis of morphogenesis*, a mathematical framework to describe the morphogensis of an embryo using diffusion ($D_{1},D_{2}$ diffusion constants) and reaction (*R* reaction rate and f,g reaction terms)

(1) $$\frac{\partial c_{1}}{\partial t}=Rf\left(c_{1},c_{2}\right)+D_{1}\Delta c_{1}$$

(2) $$\frac{\partial c_{2}}{\partial t}=Rg\left(c_{1},c_{2}\right)+D_{2}\Delta c_{2}$$

of two morphogenes $c_{1},c_{2}$ in a medium or a cell and that, if certain conditions are fulfilled, periodic structures with similar sizes will emerge through instability. It is worth mentioning that Turing was fully aware of the fact, that his

*[…] model will be a simplification and an idealisation, and consequently a falsification. It is to be hoped that the features retained for discussion are those of greatest importance in the present state of knowledge*.

[64]

Nevertheless, he uses the language of mathematics, to develop a model capable of describing the fact that a homogeneous mixture of two morphogens is under certain conditions able to create structures and instabilities and therefore gain insight into a complex behavior.

Pelz et al. [63] use Turings model and interpret the two morphogenes $c_{1},c_{2}$ as population densities of two social groups, to describe the emergence of slums as Turing instability of different migration processes, based on the observation, that these settlements show a similar size in different cities of the world despite different cultural, economic, and topographical boundary conditions [65]. Rather than describing the emergence and development of social systems or informal settlement(s) in purely qualitative terms, as in the work of Luhmann [50] or Dovey [22] mentioned above, the approaches presented in this section offer the possibility of validation when the relevant data are available.

### 4.3. Linking Data and Concepts

The attempts described above are suitable for building models of social systems in general or informal settlements in particular. Based on Figure 2 (middle), the formulation of a qualitative model, such as Dovey’s [22], can be assigned to the step of abduction. In the following step, a concrete quantitative model, such as Pelz’s [63] can then be developed in the associated deduction step. However, how can it be tested whether this model reflects the empirical observations and is therefore suitable to model possible future scenarios?

One possible way is to understand social systems as dynamical systems. Dynamical systems evolve over time and future evolution is a function of previous state, dispersion, etc. In fact, the model described in Equations (1) and (2) already regards the two social groups as a dynamical system.

In the last decade, tremendous progress has been made in data-driven analysis of these dynamical systems. In this paper it is proposed to use a method of data-driven modeling, which has been developed in recent years based on work in fluid dynamics to describe dynamical systems [66] to link quantitative empirical observations and qualitative concepts of self-organization.

The basic idea is to identify equations from time-resolved observations that describe the dynamic behavior of the system in the best possible way, similar to the approach of Brunton and Kutz [67]. They start with the fact that the temporal behavior of an observable *u* of a system can be described as:

(3) $$u_{t}=N\left(u\left(x,t\right),u^{2},u_{x},u_{y},\ldots ,x,\xi \right)$$

with the parameter vector ξ. This equation can than be rewritten as a linear combination

(4) $$u_{t}=\xi_{1}+\xi_{2}u+\xi_{3}u^{2}+\xi_{4}u_{x}+\ldots .$$

With the observation of *n* timesteps we can construct the following over-determined linear system

(5) $$\left(\begin{array}{c} u_{t}\left(x,t_{1}\right)\\ u_{t}\left(x,t_{2}\right)\\ \ldots \\ u_{t}\left(x,t_{n}\right) \end{array}\right)=\left(\begin{array}{cccc} 1 & u(x,t_{1}) & u^{2}\left(x,t_{1}\right) & \ldots \\ 1 & u(x,t_{2}) & u^{2}\left(x,t_{2}\right) & \ldots \\ \ldots \\ 1 & u(x,t_{n}) & u^{2}\left(x,t_{n}\right) & \ldots \end{array}\right)\left(\begin{array}{c} \xi_{1}\\ \xi_{2}\\ \xi_{3}\\ \ldots \end{array}\right)=\Theta \xi$$

with the term library Θ can be constructed. The unknown parameter vector ξ can than be identified using regression methods. This framework was already used to search for equations describing the behavior of systems and identify applications in the description of a wide variety of phenomena [68]. If spatially and temporally resolved datasets on social systems, such as for example the population distribution, are now used, and the observed variable is interpreted as *u*, it is possible to investigate whether the temporal dynamics of these systems can be described using differential equations.

**Figure 4:**
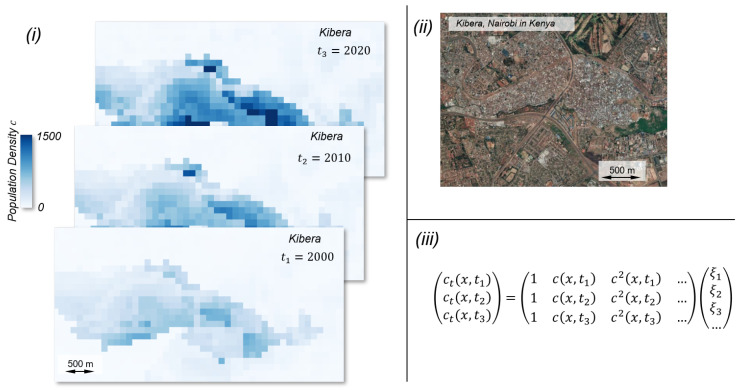
(**i**) Representation of the population distribution in Kibera, Nairobi using WorldPop data [69]. (**ii**) Satellite image of Kibera. Maps data: 2023 Google. (**iii**) Equation system to identify parameters $\xi_{i}$ to model the temporal development of the population distribution using the spatio-temporal data shown in (**i**).

In Figure 4, (i), the spatial distribution of the population distribution for the famous slum Kibera in Nairobi, Kenya is shown for three exemplary time steps (2000, 2010, and 2020). The temporal development can be calculated for different time steps, and the parameters $\xi_{i}$ can be calculated using regression methods [67].

**Figure 5:**
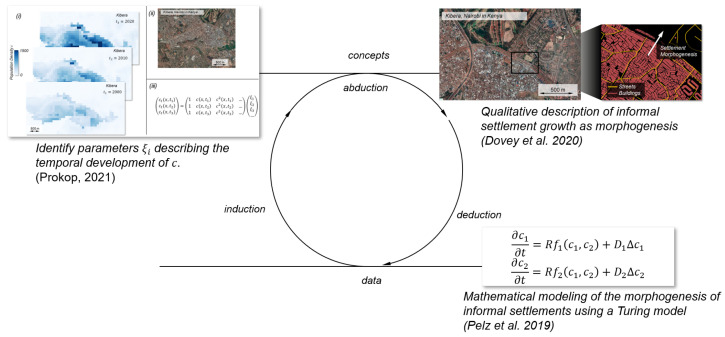
Combinationof different approaches [22,63,70] to investigate informal settlements.

It is at this point that the circle of the inferential triad proposed by Peirce described above can be closed (see Figure 5). In a general way, we can state, that the quantitative sciences have the tools (e.g., techniques for regression analysis) to investigate *the extent* to which a particular model can be represented by the data. However, the question of *which* model to examine for fitness is a qualitative one. In this approach it is the identification of a suitable term library (columns in the term library Θ).

Prokop et al. [70] already applied a similar framework to study the temporal development of regular settlement structures in countries of the Global South showing the potential of these methods to study social systems. They use time-resolved population data (WorldPop) [69] and search for differential equations that best describe the evolution of the population data.

Similar to Prokop, the temporal development of informal settlements could be investigated to determine if the population dynamics can be described as a process of self organization, like Pelz et al. [63] stated. The process is shown in Figure 5. Therefore, the population densities $c_{1}$ and $c_{2}$ can be seen as observable *u* described in Equations (3)–(5). As indicated above, based on Dovey’s observation [22] that informal settlements exhibit a kind of morphogenesis, a mathematical model for this kind of settlement can be set up [63]. In the following step, with the help of the last presented approach, it is investigated to what extent the actual temporal evolution of the settlement corresponds to the proposed model. For this purpose, the parameters $\xi_{i}$ are determined. Finally, with the help of the term library Θ and the quantitatively determined parameters $\xi_{i}$, a mathematical model for the description of the temporal development of a certain informal settlement can be set up and its quality can be evaluated by simulations. Since the system of equations found depends on the areas studied, it is still necessary to investigate the transferability to other informal settlements.

Besides, with the help of this approach there is the possibility to apply further concepts of pattern formation, morphogenesis, or self-organization to data on the development of informal settlements. One example could be Wolpert’s concept of positional information [71]. In contrast to previous approaches from data science using black box models to describe urban developments [72], with the presented framework we have an opportunity to explicitly investigate the existing dynamics in social systems by connecting qualitative concepts with data.

## 5. Discussion

This work deliberately began with a reference to the Bible. Religions are sometimes accused of referring to an absolute, objective, not verifiable truth. Similar criticism is directed towards classical social sciences, which are mostly qualitative, in comparison to quantitative (computational) social sciences. The latter are accused of taking a general “God’s eye view”. Classical social science questions whether such a view is possible at all when analyzing social and human behavior. At the same time, computational social sciences take the approach of describing and analyzing a problem based on empirical evidence, rather than limiting themselves to unverifiable general concepts.

To address major current socio-technical issues, such as upgrading informal settlements and the reality of life for a significant proportion of the world’s population, approaches combining qualitative and quantitative research are needed. On the one hand, qualitative research is essential for understanding why people move to cities and addressing their motivations.. On the other hand, a purely qualitative analysis is not sufficient, since concrete scenarios with a quantitative basis are needed for planners and engineers, for example for the planning of infrastructure provision [73].

The basic question that must be answered is the purpose of the respective model. George Box once formulated the following sentence: *All models are wrong, some are useful* [33]. In both qualitative and quantitative modeling of social issues, such as the development of informal settlements described here, the question to be answered with the help of the model should be formulated as concretely as possible. The more concretely the question can be formulated, the more likely it is that a common language can be found to bring together different perspectives.

As discussed above, the proposal to combine quantitative and qualitative methods, as in the concept of triangulation, is not new but common practice in the social sciences. Rather, the approach proposed here aims to bring different scientific disciplines more into conversation with each other by using the language of mathematics in order to leverage the full potential of different model conceptions. While Dovey’s morphogenesis of informal settlements proposes a qualitative concept that contributes to a deeper understanding of this form of settlement, the approach presented here can be used to formulate this concept with the language of mathematics. These quantitative models can then form the basis of further use cases, such as scenario development for infrastructure planning.

Nudzor has rightly pointed out that an integrated view combining qualitative and quantitative perspectives in the social sciences, such as the one presented in this paper, cannot be the panacea for solving all sociological research questions [74]. Some conceptual questions are not suitable for quantitative sciences, while for example statistical analyses cannot be performed using qualitative methods. Nevertheless, the presented framework offers a possibility to combine different perspectives in order to arrive at new approaches and models.

However, it must always be kept in mind that the language of mathematics can be a hurdle in the communication of different researchers. The advantage of the precise formulation of problems, which Richardson mentions in the above quotation, can become a disadvantage in interdisciplinary discussions, since this formulation does not take into account the range of human sensibilities from the point of view of qualitative researchers.

Nevertheless, the suggestion made above to view social systems as dynamic systems and to use data-driven approaches to search for appropriate models to describe them fulfills, on the one hand, the request of the social sciences for simpler models [34]. At the same time, it offers the possibility of deriving theses, investigating them mathematically, and developing scenarios on their basis. The framework gives the opportunity to connect the ever increasing amount of data on sociological questions with different models, check their fit, and create new models.

## 6. Conclusions

Both qualitative and quantitative approaches are employed to study social systems, with each approach having its own advantages and disadvantages. Qualitative approaches lead to a deeper understanding of social systems, while quantitative approaches enable the drawing of concrete conclusions for decision makers.

In this paper, a proposal has been made to combine the two perspectives. In doing so, qualitative work and concepts serve as the basis for understanding a phenomenon, with this understanding being extended to include mathematical models. The fit of these models is then explored using data-driven approaches, which are commonly used to analyze physical processes. This creates the opportunity to mathematically model well-known sociological concepts, such as Luhmann’s autopoesis of social systems, and to test their applicability.

This approach is particularly advantageous for questions that are the subject of research in different disciplines, as demonstrated using the example of informal settlements. While social scientists are primarily interested in understanding the causes, processes, and consequences of this type of settlement for urbanization, urban planners and engineers are concerned with how planning processes, such as infrastructure, can be designed to supply this population considering different future scenarios.

This type of research, represented by the inferential triad, which according to Peirce consists of a combination of abduction, deduction, and induction, is one way to ensure greater interconnectedness of qualitative and quantitative sciences.

## Data Availability

All data used (e.g., to create figures) is cited in the appropriate places in this paper.
